# Seek and destroy: Development of novel viral therapy for EGFR-expressing tumors

**DOI:** 10.1016/j.omton.2024.200776

**Published:** 2024-02-27

**Authors:** Christine O’Connor, Justin D. Lathia

**Affiliations:** 1 Department of Infection Biology, Lerner Research Institute, Cleveland Clinic, Cleveland, OH, USA; 2 Department of Molecular Medicine, Cleveland Clinic Lerner College of Medicine of Case Western Reserve University, Cleveland, OH, USA; 3 Case Comprehensive Cancer Center, Cleveland, OH, USA; 4 Department of Cardiovascular & Metabolic Sciences, Lerner Research Institute, Cleveland Clinic, Cleveland, OH, USA; 5 Rose Ella Burkhardt Brain Tumor and Neuro-Oncology Center, Cleveland Clinic, Cleveland, OH, USA

## Main text

A hallmark of many cancers is amplification of growth factor receptors, including epidermal growth factor receptor (EGFR), which facilitates sustained proliferation. This has served as the basis for the development of inhibitors against these receptors, although targeting them in some cancers, such as glioblastoma (GBM), is challenging due to barriers, including achieving effective therapeutic concentrations in the brain and resistance through signaling network redundancy.^1^ Compounding these challenges, current conventional therapies fail to distinguish between tumor and normal cells and therefore could damage healthy, non-transformed tissue. Oncolytic viruses, therefore, represent a targeted treatment, as these engineered viruses are manipulated to selectively target tumor, not healthy, cells. Additionally, oncolytic viruses represent a self-expanding therapy, as their entry and replication into tumor cells facilitate production of newly synthesized oncolytic viral particles, capable of infecting additional tumor cells for their destruction. This “seek-and-destroy” approach effectively spreads throughout the tumor, killing tumor cells while leaving normal, healthy cells untouched.

**Figure 1:**
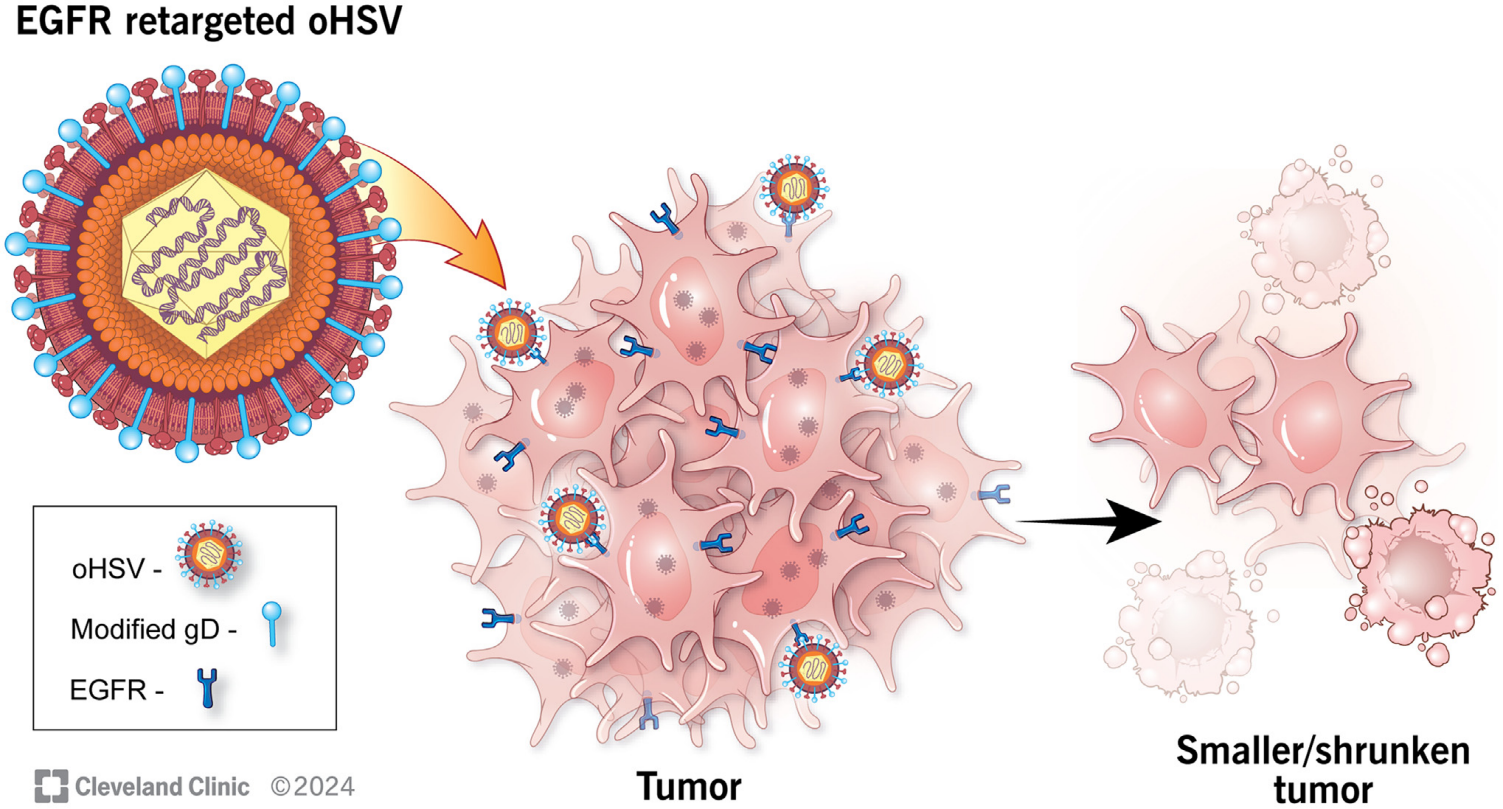
EGFR-retargeted oHSVs show reduction in tumor size

In the current study, Ingusci et al. designed new and improved oncolytic herpes simplex viruses (oHSVs) to target and treat EGFR-expressing tumors (Figure 1).^2^ oHSVs are genetically engineered, attenuated versions of the human pathogen HSV-1, designed to target, replicate in, and kill the targeted tumor cells.^3^ Investigators have pursued several HSV-encoded genes for this purpose^4^; herein, Ingusci et al. modified the HSV glycoprotein gD,^2^ which binds several cellular receptors,^5^ making this glycoprotein important for viral entry into host cells. Thus, to design effective oHSVs, the authors altered the native binding properties of gD by replacing amino acid sequences with those that recognize and bind other cell surface proteins, like EGFR. The re-engineered oHSVs retargeted for EGFR-expressing tumors display significant improvements in oHSV entry, spread, *in vitro* cell killing, and *in vivo* tumor size using a flank tumor model for GBM.^2^ These retargeted oHSVs are promising, and future work using additional GBM models to assess delivery into the brain and the impact on overall survival will prove useful.

HSV is an attractive virus for this approach, as the viral particle contains an envelope studded with viral glycoproteins that direct attachment to and entry into cells via host-encoded receptors. Thus, a clever approach to designing an effective oHSV capable of targeting tumor cells is to re-engineer the glycoprotein’s ability to interact with host cell receptors. Prior work from this group showed that HSV gD was a good candidate for modification toward an effective oHSV.^6^ HSV gD is essential for viral binding and entry; gD engages host cell receptors, leading to gH/gL activation, collectively triggering gB-mediated viral membrane fusion with the cell.^7^ gD engages several host cell receptors, including nectin-1, herpes virus entry mediator (HVEM), and a modified form of heparan sulfate, 3-O-sulfated heparan sulfate.^5^ To successfully generate an oHSV using a modified gD, this glycoprotein’s interaction with its cognate cellular receptors must be modified. To this end, the Glorioso Lab generated an oHSV, whereby a 248 amino acid (aa) single-chain variable fragment (scFv) recognizing EGFR or the EGFR variant commonly expressed in tumors, EGFRvIII, was engineered into gD in place of its native amino acids (residues 2–24) that would otherwise bind HVEM. An additional tyrosine-to-cysteine mutation at gD position 38 additionally abrogated binding to nectin-1. These alterations, along with entry-enhancing mutations in gB, resulted in enhanced entry of the retargeted oHSV.^6^^,^^8^ A concern with this approach was the potential for reversion of nectin-1-independent binding to one that is dependent. To this end, the group deleted residue 38.^8^ However, the tumor cell killing efficacy for this newer oHSV still required improvement.

In the current study, Ingusci and colleagues directed their efforts toward improved oHSV retargeting with EGFR/EGFRvIII ligands, using both affibody and variable domain from heavy-chain antibodies (VHH) approaches. A notable difference between these two new approaches versus the original scFv insertion is size; the affibody is 58 aa, and the three VHH molecules tested are between 124 and 130 aa.^2^ Like their original approach,^6^^,^^8^ the group focused on the HVEM binding region, making several recombinants spanning positions 2–24 (Δ2–24, Δ6–24, Δ7–24) for insertion of the EGFR-directed ligands, where each recombinant also contains the deletion of position 38, thereby abrogating nectin-1 binding. Based on plaque size, viral protein production, and viral titers following infection of Vero cells, three VHH oHSVs (SD1Δ6–24, SD2Δ6–24, and SD3Δ6–24) and one affibody (ZEGFRΔ7–24) were moved forward for further studies.^2^

These newly generated oHSVs revealed their improvement over the older version using the scFv insertion into gD. After demonstrating entry specificity for EGFR-expressing cells, the authors showed improved lateral spread of these newly engineered oHSVs in GBM (SNB19) and adenocarcinoma (A549) cells. Each VHH and affibody oHSV displayed significantly enhanced entry compared to the older scFv oHSV construct. The authors also noted significant improvements in *in vitro* oHSV-mediated cell death in GBM (U251, SNB19) and adenocarcinoma (A549) cells when compared to previous oHSV iterations. Importantly, this improved viral-mediated cell death was also observed *in vivo*. Using a GBM flank tumor model (U251), they showed that intravenous (i.v.) administration of oHSVs resulted in a significant reduction in tumor size (Figure 1). However, as the authors acknowledge, a single administration of the better-performing oHSV (SDΔ2–24) via the i.v. route did not result in total tumor regression. To address this, the authors evaluated tumor size following additional i.v. or intratumoral (i.t.) administration of the oHSVs, which improved outcomes, including regression of the tumor. Importantly, the added i.t. administration of SDΔ2–24 resulted in complete tumor regression in the flank tumor model.^2^ These findings suggest that boosting with this oHSV via the i.t. route would significantly improve outcomes.

These authors generated new and improved oHSVs that target EGFR-expressing tumors. Paramount to understanding their efficacy is the use of additional pre-clinical models and more representative GBM models, such as low-passage patient-derived models that better represent the cellular and molecular complexity compared to high-passage cell lines (U251). In addition, intracranial models will allow for the assessment of oHSV efficacy to cross the blood-brain barrier when administered i.v. as well as for assessment in combination with standard-of-care approaches. Such studies will lay the foundation for potential clinical trials for treatment of EGFR-expressing GBMs.
